# Assessment of Public Knowledge and Awareness of Knee Osteoarthritis Among Individuals Aged 18-65 in the United Arab Emirates (UAE)

**DOI:** 10.7759/cureus.52134

**Published:** 2024-01-11

**Authors:** Sura Salahuddin Salih, Ruqaya Mustafa Alsalihi, Noura Esam Mahboub, Nuha Yasir Jasim, Reem Ali Almutawa

**Affiliations:** 1 Surgery, Dubai Medical College, Dubai, ARE

**Keywords:** knee osteoarthritis (koa), health education and awareness, observational cross-sectional study, survey, knowledge level

## Abstract

Background

Osteoarthritis (OA) is a disease characterized by chronic degeneration of articular cartilage and subchondral bone and inflammation of the synovium, leading to pain and joint disability. The best-known risk factors for OA are increasing age and obesity. Public knowledge of this disease should be assessed as OA, given its high prevalence, which places a significant burden on patients' daily activities and their overall quality of life.

Methodology

This cross-sectional study was conducted between September and November 2023 among the United Arab Emirates (UAE) population using an online survey form. The survey responses were then analyzed using the SPSS Statistics system (IBM Corp. IBM SPSS Statistics for Windows. Armonk, NY: IBM Corp).

Results

A total of 363 people participated in this study. The sample showed a high level of knowledge of the underlying mechanism of OA, with 233 (64.2%) correctly identifying the cause. In addition, most of the participants, 244 (67.2%), were aware that OA is a chronic disease, and 243 (66.9%) knew that it is not a rare disease. Moreover, 288 (79.6%) could identify that a high BMI is a major risk factor for OA. Total and partial joint replacement for advanced disease remain the mainstay of treatment. In this study, 241 (66.4%) participants knew that joint replacement surgery was the final option to relieve the symptoms of OA. The total knowledge level of the participants showed that only 74 (20.4%) had good knowledge, 91 (25.1%) had average knowledge, and a majority of 198 (54.5%) had poor knowledge. Our study showed that a higher level of education leads to a better understanding and awareness of OA, which is generally expected (p=0.019). In addition, trust in healthcare and study material as a source of information on the topic significantly impacts knowledge, in contrast to trust in relying on family, media, and personal history (p<0.001).

Conclusion

OA severely limits daily activities, and the prevalence of OA is anticipated to double in the next decade. In this study, we aimed to determine the level of public knowledge about knee OA. The results indicate that the level of knowledge among individuals aged 18 to 65 in the UAE is low. Accordingly, raising awareness among the public will result in early detection of the disease, clearing misconceptions, and controlling dangerous and unproven procedures for the treatment of OA.

## Introduction

Osteoarthritis (OA) is the most common chronic joint disease worldwide [1]. In 2019, 528 million people worldwide suffered from OA, an increase of 113% since 1990 [2]. In fact, OA is the third most promptly rising condition after diabetes and dementia worldwide [3]. Anatomically, the knee joint consists of opposing bones that are covered by a shielding, firm material called cartilage, which ensures smooth contact between the bones while minimizing the risk of damage to the ends of the bones. The bones and cartilage are enclosed in a cavity lined by the synovium, a layer that produces fluid for lubrication and nutrition [4]. It is characterized by chronic degeneration of the articular cartilage and subchondral bone and inflammation of the synovium, leading to pain and joint disability [5]. The best-known risk factors for OA are increasing age and obesity. About 73% of people living with OA are older than 55 years [2]. As the average life expectancy of people is increasing in modern times due to improved healthcare, more cases of OA are being diagnosed [6]. According to statistics, the average age at diagnosis of knee OA is 55 years, so people are suffering from the disease for about 30 years longer [7]. In addition, the westernization of diets and increasing reliance on high-calorie but low-quality meals are leading to skyrocketing BMI levels in the population and morbid obesity, which poses a risk to weight-bearing joints both directly through mechanical forces and indirectly through metabolic interactions [8]. Physical inactivity and knee injury both contribute to the development of OA, with the former weakening and reducing joint stability and the latter causing direct injury to joint structures [9]. Public knowledge of this disease should be assessed, as OA places a significant burden on patients' daily activities and overall quality of life given its high prevalence. Measures should be taken to diagnose cases earlier, slow progression, and prevent further damage. OA causes significant economic loss through increased absenteeism and loss of productivity, which is a burden on community development. Therefore, early recognition of symptoms by the patient and seeking medical attention is a key element in the prognosis of the disease. This can only be achieved through adequate knowledge of OA.

This study aims to be a reliable measure of knowledge about knee OA that will enable researchers and clinicians to assess public knowledge and highlight the misconceptions and gaps that require targeted educational efforts to address these aspects and improve general awareness.

## Materials and methods

This is a cross-sectional study conducted among residents of the United Arab Emirates (UAE), including locals and expatriates of both sexes, aged 18 to 65 years. Using the computer program EPI Info version 7.2 (Centers for Disease Control and Prevention, Georgia, USA), a confidence interval of 95%, an estimated poor knowledge (ref), a gap of 64.2%, and a margin of error of 5%, the minimum required sample size is 354. Convenience sampling was done. We collected 363 responses between September and November 2023. We used a validated structured questionnaire that was initially tested in a pilot study, whose participants were excluded from the results, and distributed electronically to the public via a link that led to an online survey form. The questionnaire was translated from English into Arabic and then back into English to ensure the quality and comprehensibility of the questions. The questionnaire was provided in both Arabic and English. Consent forms were given to the participants. The questionnaire consisted of six separate sections. It started with the demographic data of the participants and covered topics including general awareness, possible pathophysiology, risk factors, symptoms, and treatment options. The survey responses were then analyzed using the SSPS Statistics system (IBM Corp. IBM SPSS Statistics for Windows. Armonk, NY: IBM Corp.).

The knowledge scores of the OA were calculated by adding the correctly answered knowledge questions. A descriptive analysis of the data was performed. Accordingly, the overall knowledge score for each subtopic was categorized as poor if the participant scored less than 60%, average if it was between 60% and 80%, and good if the participant scored more than 80%. The results were presented graphically in diagrams and inserted into tables. In addition, cross-tabulations were used to determine whether the demographic variables had a significant influence on the level of knowledge. Chi-square two-tailed tests were used to assess the relationship with a significant p-value of 0.05 and below.

## Results

A total of 363 participants, who met the inclusion criteria, completed the study questionnaire. Most of the participants, 292 (80.4%), were females, and only 71 (19.6%) were males, of whom 164 (45.2%) were UAE nationals and 199 (54.2%) were non-UAE nationals (Table 1).

**Table 1:** Demographic data of the participants UAE: United Arab Emirates

Demographic value	Number of participants	Percentage
Gender of participants		
Female	292	80.4%
Male	71	19.6%
Age group		
18-25	161	44.4%
26-35	49	13.5%
36-45	62	17.1%
46-55	61	16.8%
55-65	30	8.3%
Nationality		
UAE national	164	45.2%
Non-UAE national	199	54.8%
Educational level		
Uneducated	5	1.4%
Primary	4	1.1%
Secondary	66	18.2%
University and above	288	79.3%
What is your source of information about osteoarthritis?		
personal history	59	16.3%
Friends/relatives	58	16.0%
Media	127	35.0%
Study	79	21.8%
Healthcare	40	11.0%
Do you know someone diagnosed with osteoarthritis?		
Yes	230	63.4%
No	133	36.6%

When examining the knowledge of general concepts among the participants, 244 (67.2%) knew that OA is a chronic problem, and 243 (66.9%) knew that knee OA is not a rare disease. In addition, 162 (44.6%) knew that all types of joints can be affected by OA. Finally, 133 (36.6%) knew that OA is not caused by cold and damp environments. Overall, 138 (38%) of the participants had good knowledge of the disease, 130 (35.8%) had average knowledge, and 95 (26.2%) had poor knowledge.

**Figure 1:**
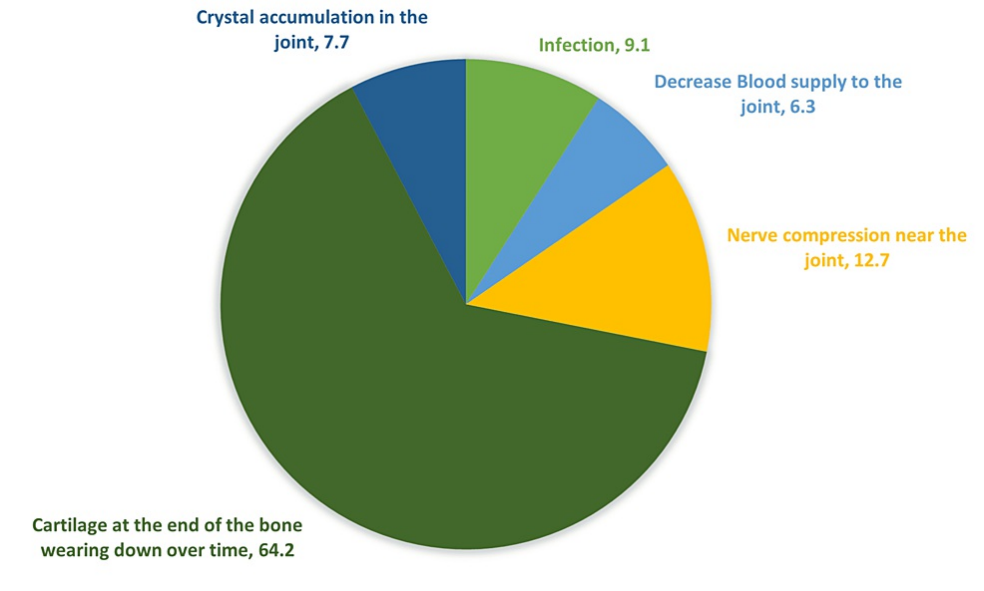
The answers of participants in percent concerning the pathophysiology of OA OA: osteoarthritis

About 233 (64.2%) showed an understanding of the basic processes of OA and correctly recognized it as a deterioration of the protective cartilage at the bone ends over time. A small percentage, 23 (6.3%) participants, associated it with reduced blood supply to the joint, while 46 (12.7%) associated it with compression of a nerve near the joint. Thirty-three (9.1%) thought that the cause was an infection. A small proportion, 28 (7.7%), also believe it may be a crystal buildup in the joint (Figure 1).

In terms of risk factors, the majority, 293 (80.7%), knew that aging is a risk factor. Although females have a higher risk of developing OA and are afflicted more severely by the disease, only half, 182 (50.1%), of the participants knew of the gender differences associated with OA. Although a strong genetic basis for OA is recognized, only 189 (52.1%) knew about the genetic link to OA. Two hundred eighty-nine participants (79.6%) knew that increased weight puts additional stress on the joints supporting the weight, increasing the risk of OA in that joint. In general, 253 (69.7%) participants knew that exercise is the most effective non-drug treatment for pain relief and improvement of mobility in patients with OA. In addition, 218 (60.1%) participants knew that a previous joint injury increases the risk of developing OA. When assessing knowledge of risk factors, 148 (40.8%) had good knowledge, while 215 (59.2%) had poor knowledge.

In terms of signs and symptoms, 185 (51%) of our participants knew that pain was not the only symptom of OA. Almost half, 189 (52.1%), recognized stiffness as an OA symptom, and 142 (39.1%) knew that redness of the knee is a sign of knee OA. In addition, 222 (61.2%) also recognized locking of the knee as a sign of knee OA, 149 (41%) knew that increased joint warmth is a sign of knee OA, and 239 (65.8%) agreed that OA may lead to loss of joint motion. Approximately 198 (54.5%) knew that muscle weakness was a sign of knee OA; however, only 139 (38.3%) knew that numbness was also a sign of knee OA. When assessing participants' knowledge of the symptoms, the majority, 211 (58.1%), had poor knowledge, 52 (14.3%) had average knowledge, and 100 (27.5%) had good knowledge.

Regarding the management of OA, analgesics are used to relieve pain and other symptoms, which was known by more than half of the participants, 193 (53.2%). In thermotherapy, the joints are treated with heat or cold to relieve the symptoms of OA. Two hundred twenty-four (61.7%) of our participants were aware of this. About 242 (66.7%) of our participants knew that certain types of exercise, including water sports that use the buoyancy of water to reduce the load of body weight on the joints, are beneficial for patients with OA. Most of our participants, 285 (78.5%), agreed that physiotherapy can significantly improve OA. More than half, 238 (65.6%), of our participants responded that intra-articular injection of steroids may help improve OA symptoms. About 241 (66.4%) of participants knew that OA would eventually require joint replacement therapy, as this is the last resort to relieve the symptoms of OA. Regarding the level of knowledge about management, 144 (39.7%) had good knowledge, 88 (24.2%) had average knowledge, and 131 (36.1%) had poor knowledge (Table 2).

**Table 2:** Answers of the participants to the questions in the survey

Question	Yes		No		Not sure	
	Number	Percentage	Number	Percentage	Number	Percentage
Do you think that knee osteoarthritis is a chronic problem?	244	67.2%	42	11.6%	77	21.2%
Do you think that knee osteoarthritis is rare?	49	13.5%	243	66.9%	71	19.6%
Do you think that all types of joints may suffer from osteoarthritis?	162	44.6%	104	28.7%	97	26.7%
Do you think that knee osteoarthritis is the result of a cold or moist atmosphere?	100	27.5%	133	36.6%	130	35.8%
Do you think that genetics is a risk factor for osteoarthritis?	189	52.1%	94	25.9%	80	22.0%
Do you think that advancing age is a risk factor for osteoarthritis?	293	80.7%	36	9.9%	34	9.4%
Do you think that high body mass index (a measure of body fat based on height& weight) is a risk factor for knee osteoarthritis?	289	79.6%	23	6.3%	51	14.0%
Do you think that the incidence of knee osteoarthritis is equal between males and females?	94	25.9%	182	50.1%	87	24.0%
Do you think that being active makes osteoarthritis feel better?	253	69.7%	51	14.0%	59	16.3%
Do you think that a previous knee injury is a risk factor for osteoarthritis?	218	60.1%	56	15.4%	89	24.5%
Do you think that pain is the only symptom of knee osteoarthritis?	119	32.8%	185	51.0%	59	16.3%
Do you think joint stiffness is a sign of knee osteoarthritis?	189	52.1%	57	15.7%	117	32.2%
Do you think knee redness is a sign of knee osteoarthritis?	142	39.1%	116	32.0%	105	28.9%
Do you think locking of the knee is a sign of knee osteoarthritis?	222	61.2%	54	14.9%	87	24.0%
Do you think increased joint warmth is a sign of knee osteoarthritis?	149	41.0%	96	26.4%	118	32.5%
Do you think that osteoarthritis may lead to loss of joint movement?	239	65.8%	52	14.3%	72	19.8%
Do you think weakness of the muscle is a sign of knee osteoarthritis?	198	54.5%	75	20.7%	90	24.8%
Do you think numbness is a sign of knee osteoarthritis?	139	38.3%	120	33.1%	104	28.7%
Do you think that analgesics (painkillers) may improve symptoms?	193	53.2%	107	29.5%	63	17.4%
Do you think that hot or cold packs to the knee may improve symptoms?	224	61.7%	75	20.7%	64	17.6%
Do you think that some types of exercises, such as swimming, are suitable for osteoarthritis patients?	242	66.7%	46	12.7%	75	20.7%
Do you think that physiotherapy can improve the symptoms of osteoarthritis?	285	78.5%	38	10.5%	40	11.0%
Do you think that steroid injections (hormones that reduce inflammation, e.g., cortisone) in the joint help to relieve severe symptoms temporarily?	238	65.6%	33	9.1%	92	25.3%
Do you think that joint replacement surgery will be needed at some point for osteoarthritis?	241	66.4%	33	9.1%	89	24.5%

**Figure 2:**
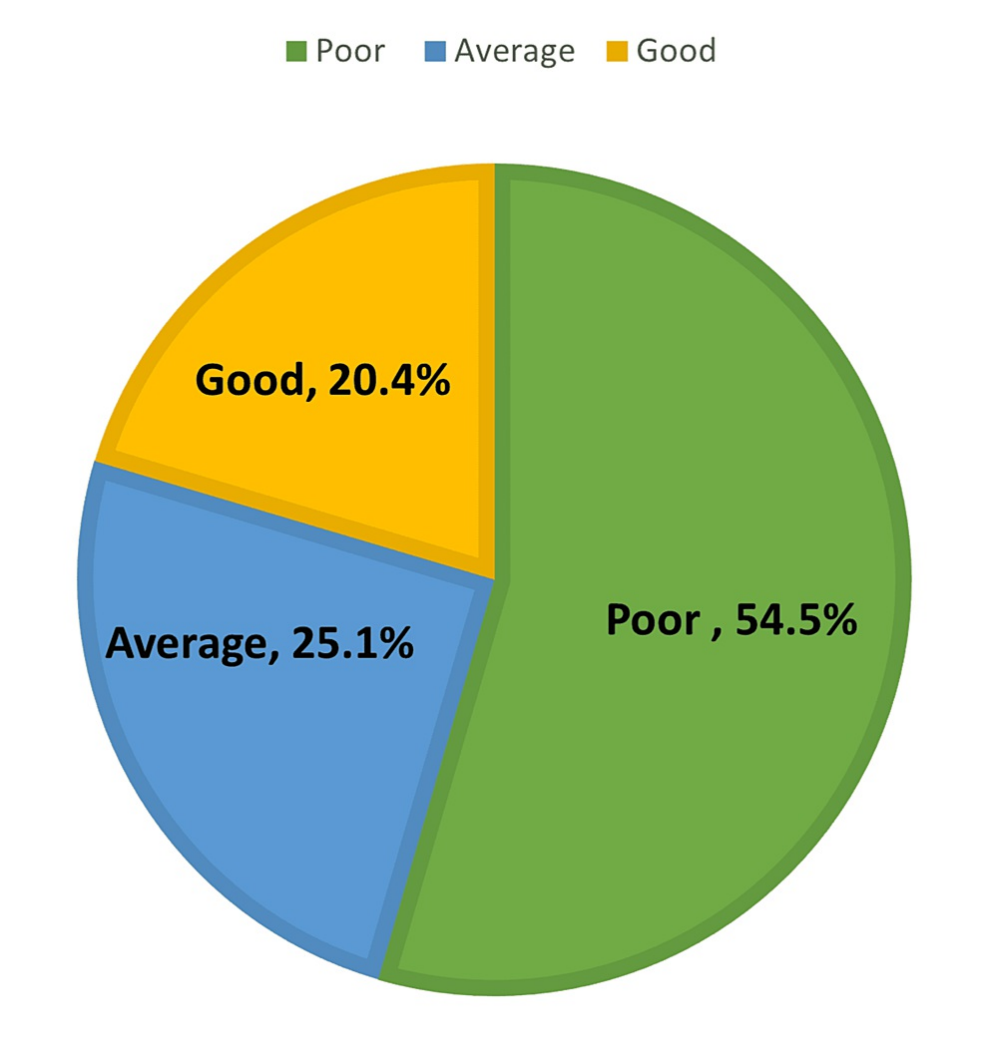
The total knowledge level of the participants according to their total score

The overall knowledge level of the participants showed that only 74 (20.4%) had good knowledge, 91 (25.1%) had average knowledge, and the majority, 198 (54.5%), had poor knowledge (Figure 2).

We investigated whether demographic changes in age, gender, nationality, or education level had an impact on the overall level of knowledge. Among females, 154 (52.7%) had a poor level of knowledge, 76 (26.0%) had an average level, and 62 (21.2%) had a good level. In contrast, 44 (62.0%) males had a poor level of knowledge, 15 (21.1%) had an average level, and 12 (16.9%) had a good level. However, this difference is not significant (p=0.374). No statistically significant difference in the level of knowledge was found between the different age groups (p=0.525). In the 18-25 age group, 81 (50.3%) have a poor level of knowledge, 42 (26.1%) have an average level, and 38 (23.6%) have a good level. In the 26-35 age group, the majority, 30 (61.2%), have a poor level of knowledge, while 10 (20.4%) have an average and nine (18.4%) have a good level of knowledge. In the 36-45 age group, 31 (50%) have a poor level of knowledge, 17 (27.4%) have an average level, and 14 (22.6%) have a good level. In the 45-55 age group, 37 (60.7%) have a poor level of knowledge, 17 (27.9%) have a medium level, and seven (11.5%) have a good level. Finally, in the 55-65 age group, 19 (63.3%) have a poor level of knowledge, five (16.7%) have an average level, and six (20%) have a good level. Among non-UAE nationals, 102 (51.3%) have a poor level of knowledge, 49 (24.6%) have an average level of knowledge, and 48 (24.1%) have a good level of knowledge. In contrast, 96 (58.5%) UAE nationals have a poor level of knowledge, 42 (25.6%) have an average level of knowledge, and 26 (15.9%) have a good level of knowledge. Therefore, no significant difference was found (p=0.141). However, the analysis revealed a significant difference between the educational levels (p=0.19). The level of knowledge in the uneducated category shows that three (60%) of the participants fall into the poor category and two (40%) into the average category. At the primary level, four (100%) of the participants in this category had poor knowledge of knee OA. At the secondary level, 47 (71.2%) have poor knowledge, 12 (18.2%) have average knowledge, and seven (10.6%) have good knowledge about OA. Among participants with a university degree and above, 144 (50%) participants have a poor level of knowledge, 77 (28.7%) have an average level of knowledge, and 67 (23.3%) have a good level of knowledge. The source of information had the greatest influence on the level of knowledge (p>0.001). Among those who obtained their information from the healthcare system and study material, the number of people with poor knowledge was the lowest at 15 (37.5%) and 26 (31.3%), respectively, while the number of participants with good knowledge was the highest at 15 (37.5%) and 26 (31.3%), respectively. Friends and relatives, media, and personal history were not good sources of information, as those with these sources scored the lowest in knowledge of OA, with 86 (66.7%), 33 (63.5%), and 38 (64.4%) scoring poorly and only 17 (13.2%), 6 (11.5%), and 10 (16.9%) scoring well. Knowing someone with OA had no significant effect on the level of knowledge (p=0.385), with more than half of the participants, 122 (53%), having a poor level of knowledge on this topic (Table 3).

**Table 3:** Factors affecting the total knowledge levels of the participants UAE: United Arab Emirates

Factor	Awareness level						P-value
	Poor	%	Average	%	Good	%	
Gender							
Female	154	52.7%	76	26.0%	62	21.2%	0.374
Male	44	62.0%	15	21.1%	12	16.9%	
Age in years							
18-25	81	50.3%	42	26.1%	38	23.6%	0.525
26-35	30	61.2%	10	20.4%	9	18.4%	
36-45	31	50.0%	17	27.4%	14	22.6%	
46-55	37	60.7%	17	27.9%	7	11.5%	
56-65	19	63.3%	5	16.7%	6	20.0%	
Nationality							
Non-UAE national	102	51.3%	49	24.6%	48	24.1%	0.141
UAE national	96	58.5%	42	25.6%	26	15.9%	
Educational level							
Uneducated	3	60%	2	40%	0	0%	0.019
Primary	4	100%	0	0%	0	0%	
Secondary	47	71.2%	12	18.2%	7	10.6%	
University and above	144	50.0%	77	26.7%	67	23.3%	
Source of information							
Friends/relatives	86	66.7%	26	20.2%	17	13.2%	<0.001
Healthcare	15	37.5%	10	25.0%	15	37.5%	
Media	33	63.5%	13	25.0%	6	11.5%	
Personal history	38	64.4%	11	18.6%	10	16.9%	
Study	26	31.3%	31	37.3%	26	31.3%	
Know someone with osteoarthritis							
Yes	122	53.0%	56	24.3%	52	22.6%	0.385
No	76	57.1%	35	26.3%	22	16.5%	

## Discussion

The study investigated public knowledge of knee OA in people aged 18 to 65 years in the UAE. The study included a large sample in terms of age, gender, and education level that is representative of the general public. The study investigated the public's knowledge of OA and associated risk factors, pathophysiology, and knowledge of treatment.

Of the 363 participants, more than half, 292 (80.4%), were female, the majority, 288 (79.3%), had a university degree or more, 161 (44.4%) were between 18 and 25 years old, and 127 (35.0%) of the participants used the media as their main source of information, which is an easily accessible and simple source of information, especially for the younger population.

The sample had a high level of knowledge of the underlying mechanism of OA, with 233 (64.2%) correctly naming the cause. In addition, most participants, 244 (67.2%), were aware that OA is a chronic disease, and 243 (66.9%) knew that it is not a rare disease.

The most common symptom of OA is pain, which usually prompts a visit to the doctor. Other symptoms include stiffness, which most often occurs in the morning or after a period of inactivity and usually disappears within 20 to 30 minutes. Approximately half of our sample, 185 (51.0%), were aware that pain is not the only symptom of OA, and slightly more than half, 189 (52.1%), recognized stiffness as an OA symptom.

The majority, 293 (80.7%), were aware that aging increased the risk of OA, and only 182 (50.1%) were aware of gender differences. A strong genetic basis for OA is a known risk factor, and heredity accounts for about half of OA cases. In addition, 289 (79.6%) were able to recognize that a high BMI is an important risk factor for OA.

Total and partial joint replacement in advanced disease remains the most important treatment option. In this study, 241 (66.4%) of the participants knew that joint replacement surgery was the last option to alleviate the symptoms of OA.

The differences between genders, nationalities, and age groups did not significantly affect the level of knowledge. However, our study showed that higher levels of education and certain sources of information, namely healthcare and study, have a greater understanding and awareness of OA, which is generally expected.

Compared to another study conducted in Jeddah, Saudi Arabia, our study had a higher percentage of female participants: 292 (80.4%) in our study compared to approximately 681 (55%) in Jeddah. In addition, the age group between 18 and 25 years was predominant in both studies, and about 60% of the respondents in both studies-230 people in our study and about 780 in Jeddah-had a family member or friend who had OA. In Jeddah, relatives and friends were the main sources of information about OA, while in our study, the main source of information was the media. The majority of participants in both studies had a bachelor’s degree: 288 (79.3%) participants in our study and 768 (62%) in theirs, with education level having a significant impact on overall scores in both studies. No significant differences were found when comparing the results of males and females or age groups in both studies. When comparing the overall level of knowledge, both studies indicate a low level of knowledge about OA in the population of Jeddah and among individuals aged 18-65 years in the UAE [10].

Limitations

The small sample size was one of the main limitations of our study. Therefore, the results may not be representative of the population studied and cannot be generalized to the entire population. In addition, more females than men responded to the survey: 292 (80.4%) and 71 (19.6%), respectively. This may lead to distortions in the results. Furthermore, 161 (44.4%) participants were between the ages of 18 and 25, which also suggests a biased sample. Socioeconomic differences should have been taken into account in the questionnaire, as they strongly influence access to healthcare and therefore knowledge.

## Conclusions

OA severely limits activities of daily living, and the prevalence of OA is expected to increase in the coming decades as it is directly related to obesity. Patients with OA seek medical attention due to pain and stiffness, the two main symptoms of knee OA. In this study, we wanted to determine the public's level of knowledge about knee OA. The results indicate that the level of awareness is low among people aged 18 to 65 in the UAE. Accordingly, raising public awareness will lead to early detection of the disease, eliminate misconceptions, and curb dangerous and unproven procedures in the treatment of OA. This study is beneficial for individuals as they will improve their views and correct their practices toward OA, and it is beneficial for the community level as it guides awareness approaches. Therefore, we recommend a better emphasis on this condition through targeted educational methods, as it affects a significant number of individuals. However, further studies are needed across the region to investigate the level of knowledge of the population and provide evidence to clinicians of known misconceptions.
